# The effect of feature normalization methods in radiomics

**DOI:** 10.1186/s13244-023-01575-7

**Published:** 2024-01-07

**Authors:** Aydin Demircioğlu

**Affiliations:** Institute of Diagnostic and Interventional Radiology and Neuroradiology, University Hospital Essen, Hufelandstrasse 55, 45147 Essen, Germany

**Keywords:** Feature normalization, Feature scaling, Feature selection, Radiomics, High-dimensional datasets

## Abstract

**Objectives:**

In radiomics, different feature normalization methods, such as z-Score or Min–Max, are currently utilized, but their specific impact on the model is unclear. We aimed to measure their effect on the predictive performance and the feature selection.

**Methods:**

We employed fifteen publicly available radiomics datasets to compare seven normalization methods. Using four feature selection and classifier methods, we used cross-validation to measure the area under the curve (AUC) of the resulting models, the agreement of selected features, and the model calibration. In addition, we assessed whether normalization before cross-validation introduces bias.

**Results:**

On average, the difference between the normalization methods was relatively small, with a gain of at most + 0.012 in AUC when comparing the z-Score (mean AUC: 0.707 ± 0.102) to no normalization (mean AUC: 0.719 ± 0.107). However, on some datasets, the difference reached + 0.051. The z-Score performed best, while the tanh transformation showed the worst performance and even decreased the overall predictive performance. While quantile transformation performed, on average, slightly worse than the z-Score, it outperformed all other methods on one out of three datasets. The agreement between the features selected by different normalization methods was only mild, reaching at most 62%. Applying the normalization before cross-validation did not introduce significant bias.

**Conclusion:**

The choice of the feature normalization method influenced the predictive performance but depended strongly on the dataset. It strongly impacted the set of selected features.

**Critical relevance statement:**

Feature normalization plays a crucial role in the preprocessing and influences the predictive performance and the selected features, complicating feature interpretation.

**Key points:**

• The impact of feature normalization methods on radiomic models was measured.

• Normalization methods performed similarly on average, but differed more strongly on some datasets.

• Different methods led to different sets of selected features, impeding feature interpretation.

• Model calibration was not largely affected by the normalization method.

**Graphical Abstract:**

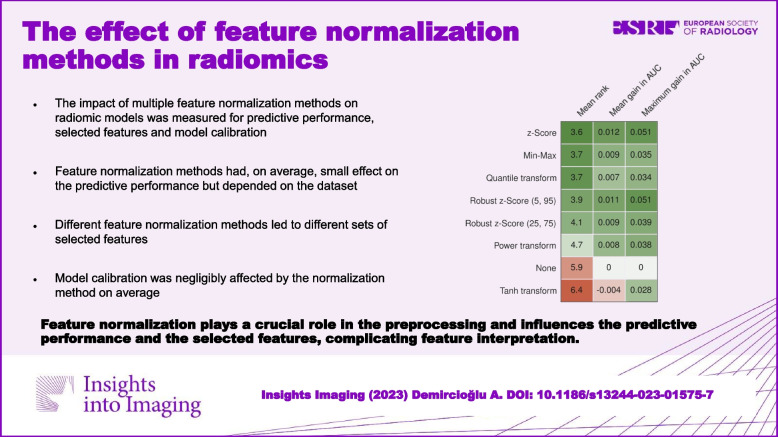

**Supplementary Information:**

The online version contains supplementary material available at 10.1186/s13244-023-01575-7.

## Introduction

Radiomics has emerged as a promising image analysis technique, providing insights for the characterization and quantification of radiological imaging and supporting diagnostic and prognostic tasks [1, 2]. Essentially, radiomics involves the application of a machine learning pipeline to process features extracted from radiological data [3–5]. The process comprises multiple steps, beginning with acquisition, segmentation, feature extraction, data preprocessing, feature selection, and classification [6].

The data preprocessing step for the extracted features primarily aims to clean the data and enhance their suitability for later processing. It encompasses various methods, such as imputing missing values, removing outliers, and harmonization [7]. An integral part of this process is feature normalization, also called feature standardization or scaling, wherein the features are scaled to balance their numerical range. Because of the diversity of radiological features extracted from imaging, which include morphological, intensity, and texture features, these features generally cannot be expected to be on similar scales. However, the presence of features on different scales could introduce bias since features with larger values might exert a more substantial influence relative to those with smaller values during subsequent feature selection and classification. It may result in more weight being erroneously given to features with larger values. Additionally, it can also lead to complications during the classifier training, as feature selection and machine learning algorithms often make implicit assumptions about the data. For instance, the presence of features with very large values might result in slow convergence in the optimizer underlying the least absolute shrinkage and selection operator (LASSO) feature selection [8] and can cause severe convergence errors in neural networks [9].

Several feature normalization methods are currently employed in radiomic studies [10]. The most prominent ones include z-Score, which scales each feature to have zero mean and a variance of one [11], and Min–Max, which linearly scales the features into the range of -1 and 1 [12]. While other normalization methods, such as quantile and power transformation, exist [13], they were not widely utilized in radiomics [10]. Despite the importance of the feature normalization method, the effect of different normalization methods is currently unclear. The extent to which the feature normalization method affects the predictive performance of the classifier is uncertain, leaving the question of whether one method could lead to better-performing models unanswered. Moreover, it is unknown whether features normalized differently can impact the feature selection method and, therefore, could lead to a change in the set of selected features.

Therefore, in this study, we aimed to measure the effect of different normalization methods on the overall predictive performance and the feature selection methods.

## Methods

We employed only previously published and publicly accessible datasets for which the corresponding ethical review boards had already approved. The ethical approval for this study was waived by the local Ethics Committee (Ethik-Kommission, Medizinische Fakultät der Universität Duisburg-Essen, Germany) due to its retrospective nature. This study was performed following the relevant guidelines and regulations.

### Datasets

A total of 15 publicly available radiomic datasets were collected for this study (Table 1). Only datasets consisting of features extracted in tabular form were included. All datasets were high-dimensional, meaning there were more features than samples, except for two datasets, Carvalho2018 and Saha2018.

**Table 1:** Overview of the datasets

Dataset	*N*	*d*	Modality	Tumor type	DOI
Arita2018	168	685	MRI	Brain	10.1038/s41598-018–30273-4
Carvalho2018	262	118	FDG - PET	NSCLC	10.1371/journal.pone.0192859
Hosny2018A	293	1005	CT	NSCLC	10.1371/journal.pmed.1002711
Hosny2018B	211	1005	CT	NSCLC	10.1371/journal.pmed.1002711
Hosny2018C	183	1005	CT	NSCLC	10.1371/journal.pmed.1002711
Ramella2018	91	243	CT	NSCLC	10.1371/journal.pone.0207455
Saha2018	922	530	DCE-MRI	Breast	10.1038/s41416-018–0185-8
Lu2019	213	658	CT	Ovarian cancer	10.1038/s41467-019–08718-9
Sasaki2019	138	588	MRI	Brain	10.1038/s41598-019–50849-y
Toivonen2019	100	7106	MRI	Prostate cancer	10.1371/journal.pone.0217702
Keek2020	273	1323	CT	HNSCC	10.1371/journal.pone.0232639
Li2020	51	397	MRI	Glioma	10.1371/journal.pone.0227703
Park2020	768	941	US	Thyroid cancer	10.1371/journal.pone.0227315
Song2020	260	265	MRI	Prostate cancer	10.1371/journal.pone.0237587
Veeraraghavan2020	150	201	DCE-MRI	Breast	10.1038/s41598-020–72475-9

*N* sample size, *d* number of features, *DOI* digital object identifier of the publication corresponding to the dataset

### Preprocessing

For this study, the features were further processed by removing non-radiomic features and merging all available data. Since a few datasets contained missing values imputation by feature mean was used prior to analysis (this concerned most notably the HosnyA, HosnyB, and HosnyC datasets, where overall 0.79%, 0.65%, and 0.19% values were missing. It affected nearly exclusively the exponential_ngtdm_contrast and exponential_glcm_correlation features, most probably due to numeric overflow). Constant features and features with more than 25% missing values were removed completely.

### Feature normalization

Seven different feature normalization methods were employed, including some commonly used in radiomics (Table 2): The z-Score, along with two robust variants of the z-Score based on interquartile ranges of (5,95) and (25,75), the Min–Max, the power, quantile, and tanh transformations. In addition, to establish a baseline, no normalization was employed in the analysis.

**Table 2:** Overview of the normalization methods, their parameters, and the source of implementation

Method	Parameter	Implementation
z-Score	-	“StandardScaler” from Scikit-learn v1.1.2
Robust z-Score (5,95)	Quantile [5,95]	“RobustScaler” from Scikit-learn v1.1.2
Robust z-Score (25,75)	Quantile [25,75]	“RobustScaler” from Scikit-learn v1.1.2
Min–Max	Scale [-1, 1]	“MinMaxScaler” from Scikit-learn v1.1.2
Power transformation	-	“PowerTransformer” from Scikit-learn v1.1.2
Quantile transformation	-	“QuantileTransformer” from Scikit-learn v1.1.2
Tanh transformation	-	Own implementation
None	-	-

The z-Score normalization proceeds by centering the data to a mean of 0 and rescaling it to a variance of 1, ensuring that the data is standardized and comparable across different features. The robust variants first center the data using the median and subsequently scale it using the specified interquartile range, making them less sensitive to outliers. The Min–Max method linearly scales the data to -1 and 1. Furthermore, the power transformation, based on Yeo-Johnson, transforms the data monotonically to reduce skewness and improve its normality [14]. Similarly, the quantile transformation normalizes the data based on quantiles to ensure uniform distribution of the values [15] . Lastly, the tanh transformation applies the hyperbolic tangent function to scale the data to a unit range while decreasing the influence of extreme values [16]).

### Feature selection methods

Four well-performing feature selection methods were employed [17]: LASSO [18], extra trees (ET) [19], analysis of variance (ANOVA) [20], and Bhattacharyya [21]. These methods determine feature importance using distinct approaches: LASSO applies a logistic regression with an L1-regularization term to identify key features, while Extra Trees constructs multiple decision trees and employs a voting mechanism. ANOVA assesses feature importance by comparing the variances between groups and within groups, whereas Bhattacharyya calculates the similarity of features interpreted as probability distributions. Since they score each feature according to their estimated relevance, a decision had to be made on how many of the highest-scoring features should be used for the subsequent classifier. The number of selected features was chosen from among *N* = 1, 2, 4, … 32, 64. The hyperparameter C for the LASSO, which balances the model fitting and the regularization, was set to *C* = 1. For the ET, 100 trees were used.

### Classifiers

Four classifiers were utilized [22]: Naive Bayes, logistic regression (LR), kernelized SVM (RBF-SVM), and random forest (RF). The hyperparameters of each method were selected through a grid search approach. Specifically, for the LR, the regularization parameter was chosen from *C* = ^1^/_64_,^1^/_16_, ^1^/_4_, 0, 4, 16, 64, while for the random forest, the number of trees was chosen from *N* = 50, 125, 250. For the kernelized RBF-SVM, the kernel width γ was set automatically to the inverse of the number of features, and C was chosen from *C* = ^1^/_64_,^1^/_16_, ^1^/_4_, 0, 4, 16, 64.

### Training and evaluation

**Fig. 1:**
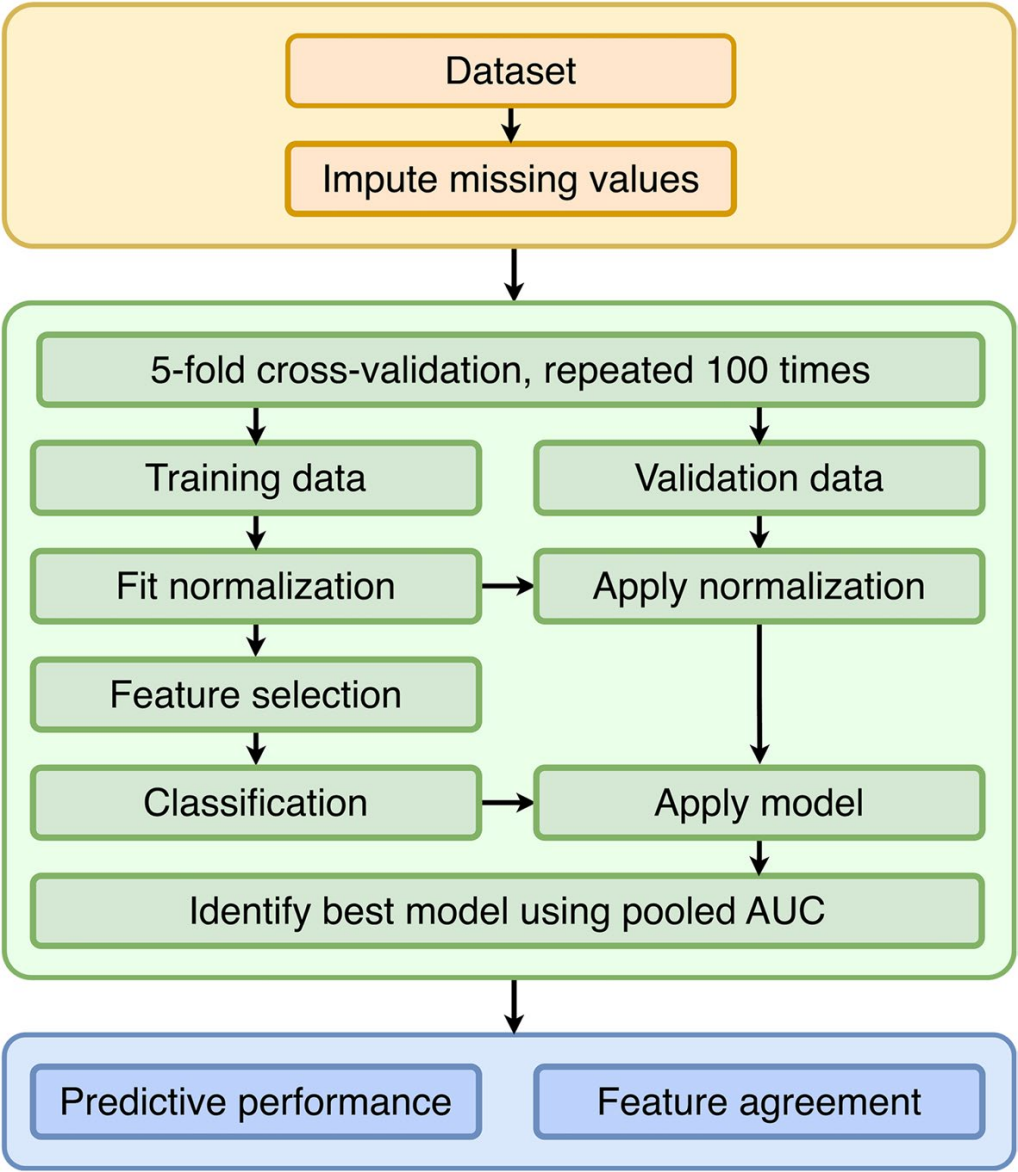
Flow diagram of the design of the experiments

Training was performed using fivefold stratified cross-validation (CV) with 100 repeats (Fig. 1). In each repeat, the data was first split randomly into five folds. Then, in turn, each fold was used once as a test fold, while the other four folds were used to determine the best-performing model using a grid search. Model training was performed by first applying a feature normalization method only to the training folds. Then, a number of selected features, a feature selection method, a classifier, and its hyperparameter were fixed, and a corresponding model was trained on the four training folds. This model was then evaluated on the left-out test fold. The resulting predictions were then pooled, and the model’s performance was then computed using AUC.

### Predictive performance

During each repeat, the predictive performance of each normalization method was evaluated by determining the best-performing model using that specific method based on the AUC. The average AUC over all repeats was then used to rank each feature normalization method. In addition, the number of datasets where a method performed best was recorded. As a secondary measure, sensitivity and specificity of the models were determined using Youden’s method [23].

### Feature agreement

Since normalization could impact which feature selection method performs best, we measured the agreement of the feature selection method of the best-performing model for each normalization method across all repeats. Even when the same feature selection method is used, feature normalization can influence the selected features. In addition, it is well-known that feature selection is unstable in data with high dimensionality [17, 24]. Therefore, we measured the agreement of the selected features across all folds, and all repeats using the Intersection-over-Union (also called the Jaccard index), which measures the degree of overlap between the selected features.

### Model calibration

Brier score [25] and expected calibration error (ECE) [26] were employed to measure the calibration of the resulting models.

### Bias from normalizing before cross-validation

To understand whether normalizing all data incorrectly before training could lead to bias, we re-run the experiment but scaled all data once up-front before splitting into folds. Accordingly, no feature normalization was applied during the CV. The predictive performance in terms of AUC was then used to compare the correct and the incorrect experiments. In addition, the differences in model calibration were measured.

### Software

All experiments were performed using Python 3.10. Normalization methods were utilized from the scikit-learn package v1.1.2 [27]. The code and data are available on github.^1^

### Statistics

Descriptive statistics were reported as mean ± standard deviation. *p* values below 0.05 were considered to be statistically significant. Statistics were computed using Python 3.10 and the scipy module. Normalization methods were compared using a Friedman test and a post hoc Nemenyi test [28].

## Results

### Predictive performance

**Fig. 2:**
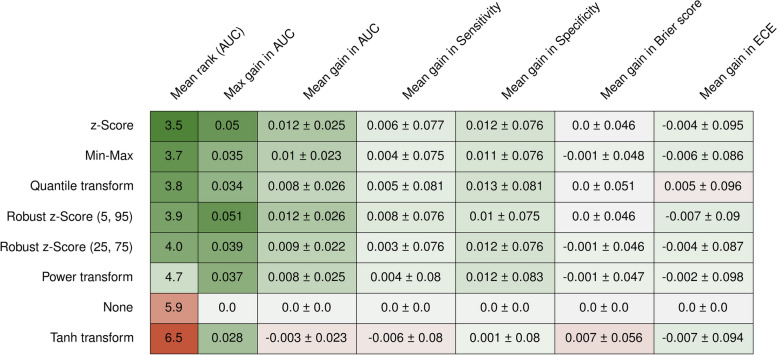
Overview of the best-performing models’ predictive performance and model calibration metrics averaged over all repeats. Numbers are reported as mean ± standard deviation

An effect of the normalization on the overall predictive performance was visible; however, on average, the gain in AUC compared to not normalizing the features was at most + 0.012, which was attained when comparing the z-Score (mean AUC: 0.707 ± 0.102) to no normalization (mean AUC: 0.719 ± 0.107) (Fig. 2; Fig. S1 in Supplementary file 1). The method that performed best across all datasets was z-Score with a mean rank of 3.5, closely followed by Min–Max, the quantile transformation, and the two robust z-Scores methods (Fig. 2). Compared to not scaling, the largest difference was + 0.051 in AUC, obtained by the robust z-Score (5,95) (mean AUC: 0.719 ± 0.107). The worst method was the tanh transformation, which, on average, performed slightly worse than not scaling (mean AUC: 0.704 ± 0.104). A slightly larger gain could be seen in the model’s specificity (around 0.01) but less so in the sensitivity of the resulting model (Fig. 2).

**Fig. 3:**
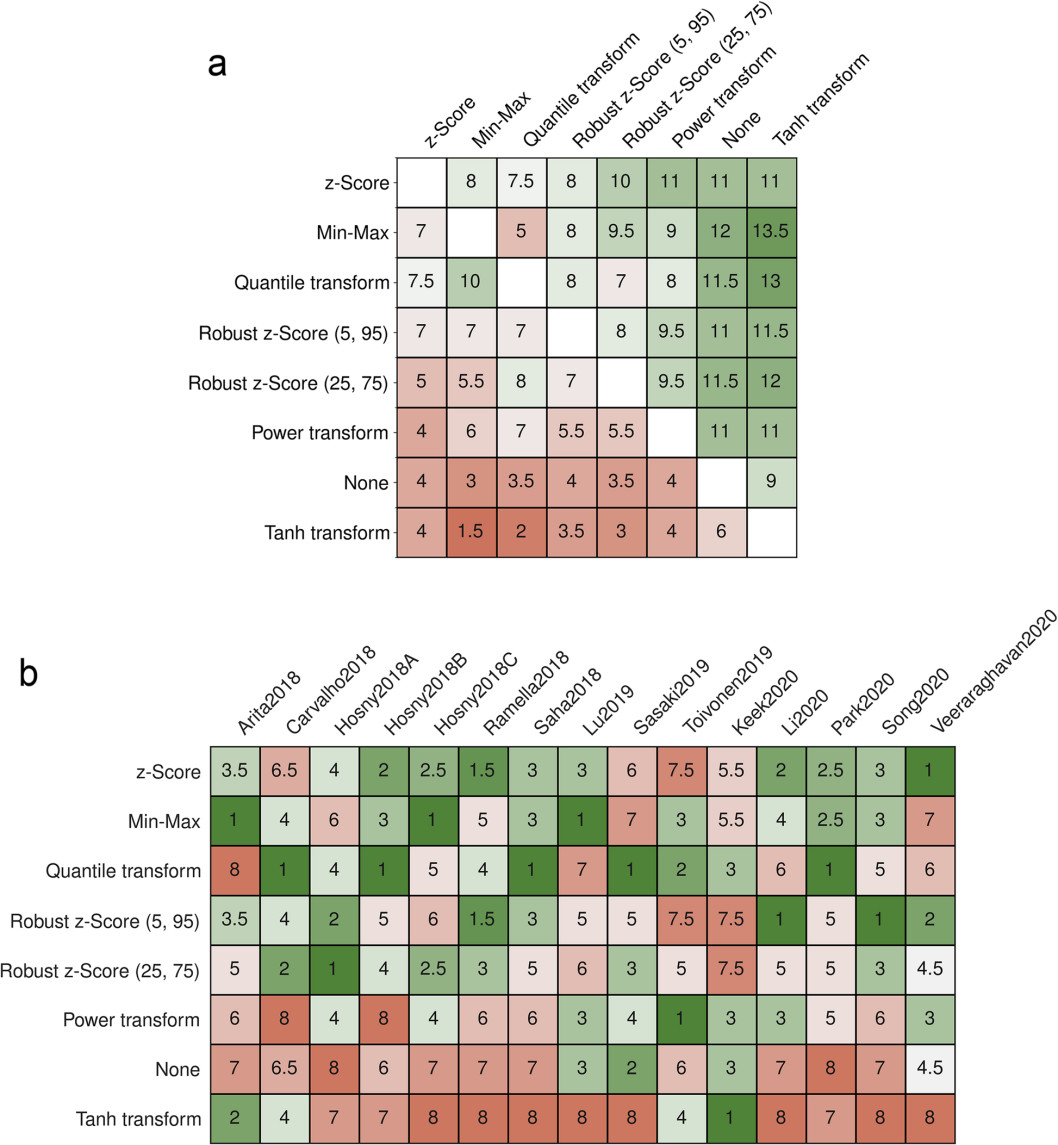
**a** Mean rank of the feature normalization methods; mean gain, and maximum gain compared to applying no normalization. **b** Counts of wins and losses between the normalization methods

Regardless, no method could consistently outperform any other. Even the best-ranked z-Score was performing in AUC lower than the worst-ranked tanh on four datasets (Fig. 3a). When considering the method that most often performed best, the quantile transformation outperformed all other methods on five datasets (5/15, 33%), while the best-ranked z-Score could only do so on one dataset (Fig. 3b).

A Friedman test indicated a significant difference between the feature normalization methods (*p* < 0.001); a post hoc Nemenyi test showed that the tanh transformation was performing significantly worse than the z-Score (*p* = 0.033), and the quantile transformation (*p* = 0.046) while no significant difference could be found for any other pair of feature normalization methods.

### Feature agreement

**Fig. 4:**
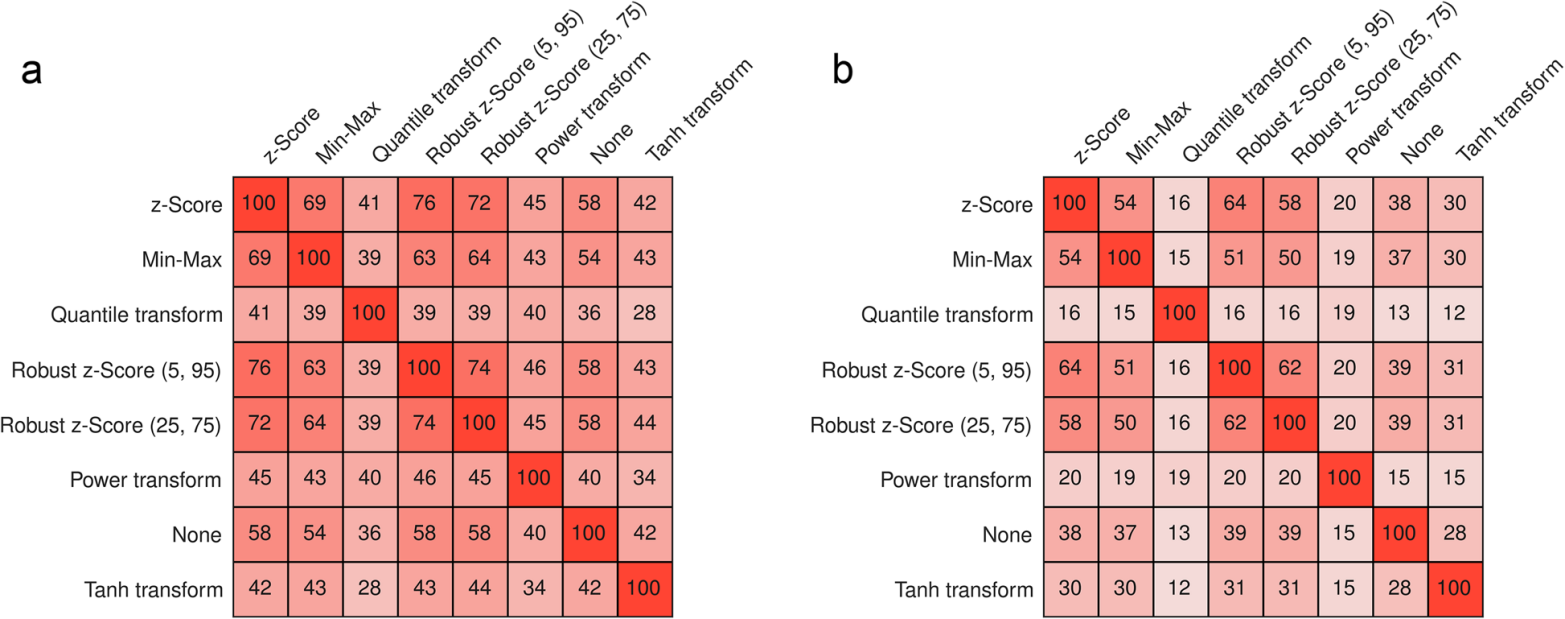
Feature agreement of the best-performing models across repeats. **a** Agreement of feature selection methods of the best-performing models (in %). **b** Feature agreement of the selected features of the best-performing models, measured via Intersection-over-Union (in %)

Feature normalization also had a strong influence on the best-performing feature selection method and the selected features (Fig. 4). The highest amount of agreement of feature selection methods was seen between the z-Score and the robust z-Score (5,95) (Fig. 4a). Regarding the selected features, lower agreements were seen (Fig. 4b). The highest agreement of selected features was between the z-Score and the robust z-Score (5,95) method with an agreement of 62%. The quantile and power transformation resulted in vastly different selected features compared to the other methods, with less than 21% agreement.

### Model calibration

Neither the Brier score nor the ECE showed large differences on average when different methods were applied (up to a loss of 0.007), suggesting that model calibration is not highly dependent on feature normalization (Fig. 2).

### Bias from normalizing before cross-validation

**Fig. 5:**
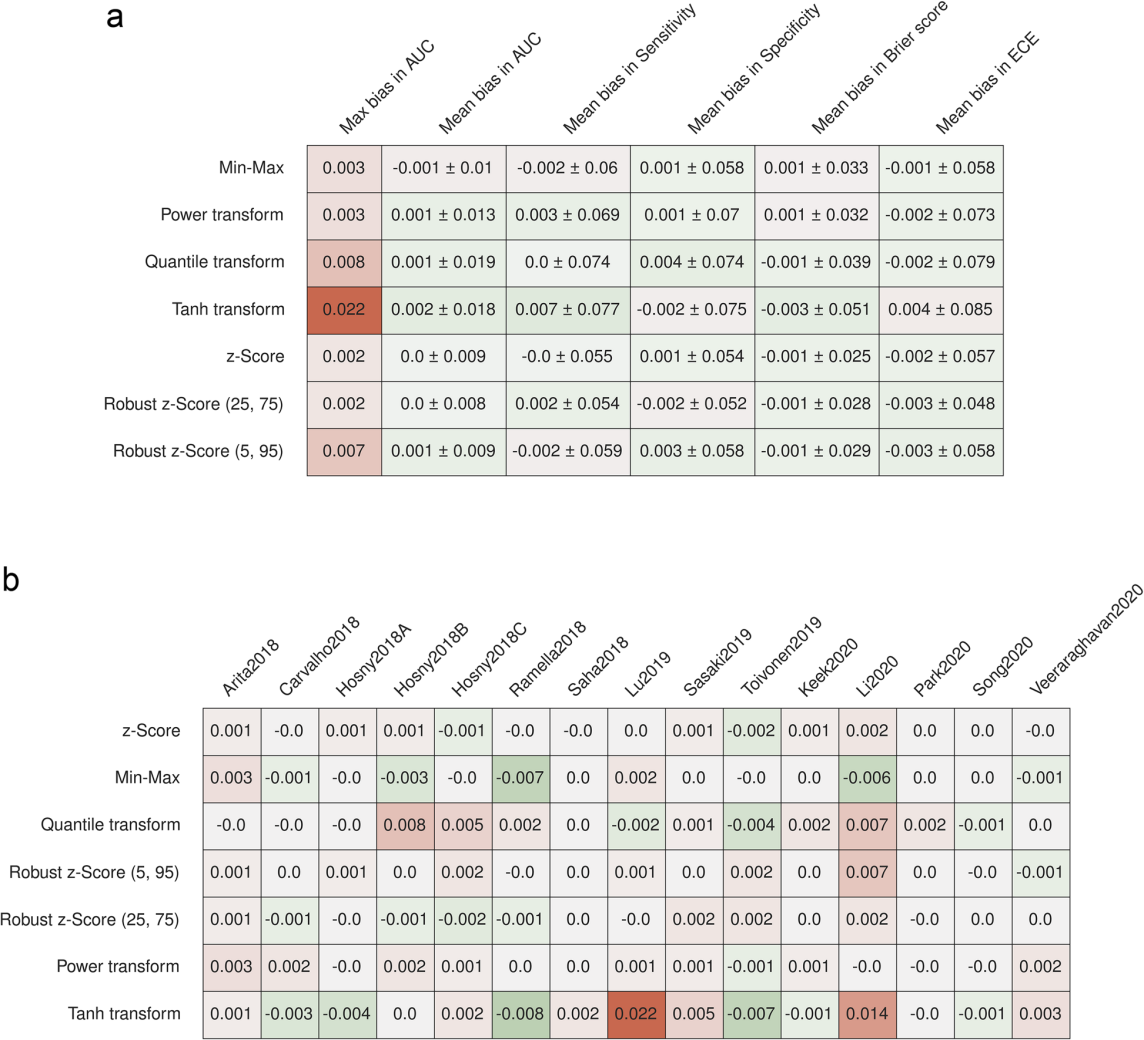
Differences of the best-performing models when applying feature normalization incorrectly before cross-validation compared to applying it correctly. **a** Differences averaged over all repeats. **b** Differences in AUC for each dataset. Numbers are reported as mean ± standard deviation

Applying feature normalization once before cross-validation did not lead to a clear bias since the mean difference in AUC was often close to ± 0.001. Only in the case of the tanh transformation a larger bias of + 0.022 was observed (Fig. 5a). On certain datasets, often differences of up to 0.01 could be seen. The largest difference was for the tanh transformation on Keek2020, where the bias reached 0.022, and on Li2020, with a bias of 0.014 (Fig. 5b). Similarly, no clear bias in the sensitivity and specificity of the resulting models and the model calibration were observed.

## Discussion

Feature normalization is a central part of the radiomics pipeline, yet its impact on the feature selection and classifier is unclear. We employed several feature normalization methods, including less commonly used methods like the power and quantile transformation, across multiple datasets to assess their influence on the predictive performance and the selected features.

Our result showed that the predictive performance depended on the feature normalization. On average, z-Score and its robust variants, Min–Max, and the quantile transformation performed best. Yet, there was no clear dominance of a single method over all others, and performance depended strongly on the dataset: for example, the quantile transformation outperformed all other methods on five datasets. In comparison, the worst-performing tanh transformation did well on two datasets, even though over all datasets it was significantly worse than the z-Score and the quantile transformation. Furthermore, the simple Min–Max method performed quite similarly to the more complex Yeo-Johnson power transformation. These results indicate that multiple methods should be tested if the goal is the highest predictive performance. A similar observation has been made in the context of feature selection methods [17, 29].

We also observed a strong effect of the feature normalization method on the feature selection. First, the normalization method impacted what feature selection method performed best. It seemingly did not depend on the predictive performance because even though the models using Min–Max and z-Score performed relatively close, the feature selection methods agreed only in about 70% across all repeats. The situation worsened when we compared the selected features across different folds. For example, the quantile transformation selected only around 15% of the same features the z-Score method did, even though both performed nearly equally well.

These observations have a very distinct impact on feature interpretation: If models were trained similarly but differed only on the feature normalization method, the conclusion on the important features can be widely different. A similar observation was already made for statistically similar performing models and is complementary to our results [30].

In contrast, the feature normalization method did not largely influence model calibration. We could not see any considerable difference in the Brier score or the ECE on average. This observation could mean that the calibration mainly depended on the classifier, not the feature normalization. However, one has to be careful since our study did not employ external datasets, where such an effect might be seen.

Surprisingly, applying feature normalization before cross-validation did not lead to a significant bias for the z-Score and the Min–Max methods. It is in stark contrast to applying feature selection before cross-validation [31]. Nonetheless, using it within the CV is advisable to avoid any risk of obtaining biased results.

Although feature normalization is known to be of importance, only a few dedicated studies have been conducted in the context of radiomics: Haga et al. considered three normalization methods, Min–Max, z-Score, and principal component analysis (PCA), in a cohort of patients with lung cancer [32]. Their results indicate that z-Score and PCA performed best, both better than Min–Max (gain in AUC of + 0.064). However, since they only consider a single dataset and a single modality (CT), one cannot deduce more general statements from this study. Yet, our results confirm their observation since z-Score performed best with a maximum gain of + 0.051. Contrary to Haga et al., we did not consider PCA since it is not commonly applied in radiomics since PCA creates new features because doing so obstructs any feature interpretation, a critical issue in radiomics.

Castaldo et al. considered multiple feature normalization methods for predicting the receptor status of breast cancer patients and stated that the normalization method does influence the resulting model [33]. They also considered the correlation of the transformed feature (compared to the original scaling). They concluded that z-Score and Min–Max do not change the features as much as transformations like quantile or whitening do. This effect can also be seen in our results because the features selected when using the (non-linear) power and quantile transformations were not similar to those selected by the (linear) z-Score or Min–Max. They also demonstrated that the best normalization method depends on the dataset, which aligns with our results, even though their dataset was also not high-dimensional since the number of features (*d* = 36) was lower than the number of samples (*N* = 91).

Other studies only considered normalization methods as part of the radiomics pipeline. Wan et al. compared three methods (Min–Max, z-Score, and mean normalization) in MRI of solid solitary pulmonary lesions and concluded that there is no large difference between them [34]. Even though they only analyzed a single dataset with a low sample size (*N* = 132), this is in line with our results. Koyuncu et al. considered two methods, Min–Max and z-Score, to detect COVID-19 in X-ray images and concluded that Min–Max performed better [35]. Even though our study showed that, on average, z-Score performs better, Min–Max performed best on three datasets, showing that Min–Max can indeed outperform z-Score. Castaldo et al. consider z-Score, quantile transformation, and a whitening method in patients with breast cancer [36]; they conclude that using quantiles performs best, although the sample size of their data is rather small (*N* = 36). Gianni et al. used different image and feature normalization methods to harmonize the features extracted from rectal MRIs [37]; they also indicated that z-Score is one of the best-performing methods.

In the machine learning context, Singh et al. compared several normalization methods and concluded that the best are z-Score and a variant called Pareto scaling, where the normalization is performed by dividing by the square root of the standard deviation [38]. Unlike our results, they stated that z-Score and its variants performed better than Min–Max. However, it has to be noted that the datasets used by Singh et al. were all low-dimensional (meaning that the number of samples exceeded the number of features) and partly synthetic. Radiomics datasets are very different in that they are nearly exclusively high-dimensional since many features are extracted, and the sample sizes are rather small. In addition, they often contain many correlated features [30]. Similarly, a recent study by de Amorim et al. considered five different normalization methods on 82 low-dimensional datasets [39]. In that study, the focus was on the interaction between the normalization and the classification methods. They concluded that z-Score normalization performed overall best, yet no single normalization method outperforms all other. This result was also observed in our study.

Based on our results, we recommend radiomic studies to test multiple feature normalization methods to obtain the highest predictive performance. If computational time is restricted, z-Score, Min–Max, and the quantile transformation should be tested.

We applied feature normalization to all features; however, in feature engineering, each feature is often normalized separately [7]. This approach is unsuitable for radiomics because of the many features involved in radiomic datasets. Yet, normalizing differently based on the type of features, i.e., morphological, intensity, or textural, and the type of image preprocessing could improve the predictive performance. In addition, our study only considered the classical radiomic pipeline; alternatives exist; for example, the feature selection might be dropped in favor of a classifier that handles feature selection implicitly, like Xgboost or random forest. Also, deep learning-based radiomics is more often used, and the features extracted from deep networks may have a different quality than those we used. In these cases, feature normalization might behave differently and should be studied in future work.

Several limitations apply to our study: We could only obtain datasets for which no external data were available (except for the two Hosny2018A and Hosny2018B datasets). However, the effect of different normalization methods on external data would be highly interesting regarding reproducibility and, therefore, the clinical applicability of the models. Given the lack of external data, we employed a simple CV with a higher number of repeats which could exhibit bias. Nested cross-validation would yield possibly unbiased results; however, it is computationally much more costly. Similarly, the AUC, sensitivity and specificity were determined using the pooled validation sets. It might be biased towards the validation set and generalize less to new data. We confined ourselves to more common normalization methods. Other methods have been defined in the literature and could be useful in the high-dimensional setting and should be tested in future studies as well.

In summary, our study has shown that, on average, feature normalization has only a minor effect on prediction performance and model calibration; however, this effect depends on the dataset. It exerts more influence on the feature selection methods.

### Supplementary Information

**Additional file 1: Figure S1.** Predictive performances of the feature normalisation methods for each dataset. **Figure S2.** Average predictive performances (AUC) of the feature normalisation methods across all datasets.

## Data Availability

All datasets are publicly available. Code, data, and results can be found on the public repository at https://www.github.com/aydindemircioglu/radNorm.

## Abbreviations

ANOVA: Analysis of variance
AUC: Area under the curve
CV: Cross-validation
DCE-MRI: Dynamic contrast enhanced MRI
ECE: Expected calibration error
ET: Extra trees
FDG-PET: Fluorodeoxyglucose-positron emission tomography
HNSCC: Head and neck squamous cell carcinoma
LASSO: Least absolute shrinkage and selection operator
LR: Logistic regression
NSCLC: Non-small cell lung cancer
PCA: Principal component analysis
RBF: Radial basis function
RF: Random forest
SVM: Support vector machine
US: Ultrasound

## Footnotes

1
https://www.github.com/aydindemircioglu/radNorm
