# Supplementary material for: The effect of feature normalization methods in radiomics
Source: Insights Imaging. 2024 Jan 7;15:2. doi: 10.1186/s13244-023-01575-7 (PMC10772134; doi:10.1186/s13244-023-01575-7)
Supplement: Supplementary file 1 — Additional file 1: Figure S1. Predictive performances of the feature normalisation methods for each dataset. Figure S2. Average predictive performances (AUC) of the feature normalisation methods across all datasets. [file 13244_2023_1575_MOESM1_ESM.pdf]

The effect of feature normalisation methods in radiomics

ELECTRONIC SUPPLEMENTARY MATERIAL

Figure S1. Predictive performances of the feature normalisation methods for each dataset.

	Arita2018	Carvalho2018	Hosny2018A	Hosny2018B	Hosny2018C
z-Score	0.818 ± 0.017	0.636 ± 0.011	0.627 ± 0.013	0.703 ± 0.015	0.618 ± 0.022
Min-Max	0.823 ± 0.017	0.637 ± 0.012	0.621 ± 0.014	0.701 ± 0.015	0.624 ± 0.023
Quantile transform	0.769 ± 0.016	0.654 ± 0.016	0.627 ± 0.014	0.709 ± 0.019	0.615 ± 0.021
Robust z-Score (5, 95)	0.818 ± 0.019	0.637 ± 0.012	0.629 ± 0.013	0.695 ± 0.012	0.613 ± 0.023
Robust z-Score (25, 75)	0.812 ± 0.019	0.638 ± 0.012	0.63 ± 0.013	0.697 ± 0.011	0.618 ± 0.022
Power transform	0.81 ± 0.019	0.635 ± 0.012	0.627 ± 0.015	0.688 ± 0.015	0.617 ± 0.022
None	0.794 ± 0.018	0.636 ± 0.01	0.618 ± 0.013	0.694 ± 0.013	0.6 ± 0.021
Tanh transform	0.822 ± 0.012	0.637 ± 0.011	0.619 ± 0.017	0.689 ± 0.01	0.595 ± 0.02

	Ramella2018	Saha2018	Lu2019	Sasaki2019	Toivonen2019
z-Score	0.766 ± 0.032	0.665 ± 0.006	0.728 ± 0.017	0.627 ± 0.022	0.823 ± 0.027
Min-Max	0.74 ± 0.029	0.665 ± 0.007	0.729 ± 0.016	0.625 ± 0.021	0.831 ± 0.022
Quantile transform	0.749 ± 0.024	0.669 ± 0.006	0.721 ± 0.015	0.648 ± 0.022	0.832 ± 0.027
Robust z-Score (5, 95)	0.766 ± 0.034	0.665 ± 0.007	0.727 ± 0.018	0.628 ± 0.022	0.823 ± 0.028
Robust z-Score (25, 75)	0.754 ± 0.031	0.664 ± 0.007	0.722 ± 0.017	0.63 ± 0.023	0.825 ± 0.026
Power transform	0.73 ± 0.029	0.662 ± 0.007	0.728 ± 0.018	0.629 ± 0.023	0.838 ± 0.03
None	0.715 ± 0.025	0.659 ± 0.008	0.728 ± 0.015	0.638 ± 0.02	0.824 ± 0.032
Tanh transform	0.699 ± 0.026	0.658 ± 0.007	0.708 ± 0.018	0.623 ± 0.022	0.829 ± 0.033

	Keek2020	Li2020	Park2020	Song2020	Veeraraghavan2020
z-Score	0.668 ± 0.01	0.872 ± 0.029	0.641 ± 0.009	0.972 ± 0.005	0.626 ± 0.027
Min-Max	0.668 ± 0.009	0.866 ± 0.031	0.641 ± 0.008	0.972 ± 0.005	0.609 ± 0.024
Quantile transform	0.671 ± 0.009	0.837 ± 0.032	0.642 ± 0.008	0.967 ± 0.005	0.611 ± 0.022
Robust z-Score (5, 95)	0.667 ± 0.01	0.876 ± 0.027	0.639 ± 0.009	0.973 ± 0.005	0.625 ± 0.026
Robust z-Score (25, 75)	0.667 ± 0.01	0.858 ± 0.031	0.639 ± 0.009	0.972 ± 0.005	0.615 ± 0.023
Power transform	0.671 ± 0.009	0.869 ± 0.022	0.639 ± 0.007	0.965 ± 0.005	0.618 ± 0.022
None	0.671 ± 0.011	0.831 ± 0.03	0.624 ± 0.01	0.964 ± 0.005	0.615 ± 0.021
Tanh transform	0.672 ± 0.008	0.808 ± 0.037	0.633 ± 0.006	0.96 ± 0.004	0.603 ± 0.02

Figure S2. Average predictive performances (AUC) of the feature normalisation methods across all datasets.

	Mean AUC
Min-Max	0.717 ± 0.107
None	0.707 ± 0.102
Power transform	0.715 ± 0.106
Quantile transform	0.715 ± 0.099
Tanh transform	0.704 ± 0.104
z-Score	0.719 ± 0.107
Robust z-Score (25, 75)	0.716 ± 0.105
Robust z-Score (5, 95)	0.719 ± 0.107
